# Classification of Chicken Carcass Breast Blood-Related Defects Using Hyperspectral Imaging Combined with Convolutional Neural Networks

**DOI:** 10.3390/foods13233745

**Published:** 2024-11-22

**Authors:** Liukui Duan, Juanfang Bao, Hao Yang, Liuqian Gao, Xu Zhang, Shengjie Li, Huihui Wang

**Affiliations:** 1 School of Mechanical Engineering & Automation, Dalian Polytechnic University, Dalian 116034, China; 13673438755@163.com (L.D.); bjf725@126.com (J.B.); yh926442612@163.com (H.Y.); glqjy9@163.com (L.G.); zhangxudlut@163.com (X.Z.); 2 School of Food Science & Technology, Dalian Polytechnic University, Dalian 116034, China; lishj@dlpu.edu.cn

**Keywords:** chicken carcass breast, defect detection, hyperspectral imaging, deep learning

## Abstract

For chicken carcass breast blood-related defects (CBDs), which occur with high frequency, the visual features are approximated in terms of the similarity of the composition of these defects, making it challenging to classify them, either manually or automatically, using conventional machine vision. The aim of this paper was to introduce a method of CBD classification based on hyperspectral imaging combined with Convolutional Neural Networks (CNNs). To process hyperspectral data, the Improved Firefly Band Selection Algorithm was constructed with the 1-D CNN CBD classification model as the objective function, achieving a reduction in the dimensionality of hyperspectral data. The multidimensional data CBD classification models were developed based on YOLOv4 and Faster R-CNN, incorporating the 1-D CNN CBD classification model and the feature fusion layer. The combination of hyperspectral data and CNN can effectively accomplish the classification of CBDs, although different model architectures emphasize classification speed and accuracy differently. The multidimensional data YOLOv4 CBD classification model achieves an mAP of 0.916 with an inference time of 41.8 ms, while the multidimensional data Faster R-CNN CBD classification model, despite having a longer inference time of 58.2 ms, reaches a higher mAP of 0.990. In practical production scenarios, the appropriate classification model can be selected based on specific needs.

## 1. Introduction

Chicken is the world’s leading consumer meat product, and chicken carcasses are one of the most important primary processed products of chicken meat [1]. The appearance of chicken carcasses is a key factor in determining their commercial value and ensuring food safety. Factors such as feed nutrition, farm management, and the slaughtering process may lead to appearance defects like bone breakage, bruising, and blood and fecal contamination, during the production of chicken carcasses [2]. The breast is the area most densely populated by chicken carcass defects, with problems including scars, abrasions, chicken breast congestion (CBC), chicken breast blisters (CBBs), and chicken breast blood residues (CBBRs) [3]. The term ‘scar’ refers to the mark left on the skin after healing from external injuries sustained during the farming process of chickens, while abrasion refers to the superficial skin damage caused by friction or scraping during transportation and slaughter. CBC refers to the collision of the slaughtered chicken carcasses with the processing equipment during dehairing and transfer, resulting in the rupture of the capillaries in the breast, which triggers the accumulation of blood in the chest cavity [4]. CBBs refer to the effects of prolonged periods of chickens lying down on their chests due to the factors of feed, management, or disease, leading to sustained pressure on the soft tissues of the chest, which in turn causes the accumulation of blood, lymphatic fluids, or other bodily fluids in the interstitial space of the chest tissues, and the eventual formation of cysts. CBBR is the blood from neck wounds sustained due to hanging from the head that is not washed off during transportation and remains on the surface of the breast after air drying. Since scars and abrasion appear as small patches of dark yellow and dark red, respectively, against the pinkish-white background of the chicken carcass, their visual characteristics are distinct, making them detectable using conventional machine vision. However, chicken carcass breast blood-related defects (CBDs), such as CBC, CBBs, and CBBRs, are primarily composed of blood, with the only differences relating to blood concentration and composition. These differences result in insignificant differences in the color and brightness characteristics of the defects in the visible wavelength range, especially if the composition is similar and the imaging characteristics are similar. Machine vision methods rely on two-dimensional spatial information such as color and luminance in the visible wavelength range for defect classification, making it difficult to effectively differentiate these defects in some cases. At present, manual inspection is mainly used to identify CBDs. The speed of manual detection can hardly keep up with the production pace, requiring 8–10 people in the defect identification process on each production line [5]. Therefore, a technology that can automatically and accurately locate and identify CBDs is needed.

Hyperspectral imaging technology is capable of creating different spectral images at different wavelengths based on differences in the chemical and physical properties of the samples [6], offering great advantage in identifying visually similar but different compositions and contents. Hyperspectral imaging technology has been widely used in food processing to detect latent defects in raw materials and finished products, such as bruising and frost damage in apples, pears, and peaches [7]. In the defect identification of chicken carcasses, hyperspectral imaging technology has similar applications, such as the identification of surface contaminants, skin diseases, and so on. After washing, fecal residues present on the surface of chicken carcasses are difficult to detect with the naked eye and traditional machine vision. Kang et al. used hyperspectral imaging technology to collect images of chicken carcasses at specific wavelengths to identify fecal contamination on the surface of chicken carcasses [8]. Skin tumors present on chicken carcasses manifest as distortions in the skin shape of the lesion area rather than discoloration, making the accuracy of identification by the naked eye and traditional machine vision relatively low. Fletcher and Kong used PCA-dimension-reduced hyperspectral images to identify skin tumors on chicken carcasses [9]. These studies indicate that hyperspectral imaging may have a unique advantage in identifying defects which have similar visual characteristics but different compositions.

Although the hyperspectral imaging system could provide valuable and rich spectral and spatial information on CBDs with approximate image characteristics, images corresponding to hundreds of hyperspectral bands also generate large amounts of redundant data [10]. These redundant data consume substantial computational resources. Processing high-dimensional data can lead to model overfitting, which may affect the accuracy of CBDs. As an emerging hyperspectral dimensionality reduction algorithm, a metaheuristic optimization algorithm is applied in hyperspectral data dimensionality reduction. These algorithms simulate natural phenomena to construct intelligent populations representing hyperspectral bands and, through the interaction and evolution of individuals, search the hyperspectral data to select the optimal spectral bands. Common metaheuristic optimization algorithms include Genetic Algorithm, Particle Swarm Optimization, Ant Colony Optimization, and Firefly Algorithm, among others [11]. A class of algorithms like the Firefly Algorithm, employing global attraction mechanisms during the search process, can cover the entire hyperspectral wavelength range, effectively avoiding the problem of local optima [12].

Even after dimensionality reduction, complex mapping is still required to utilize hyperspectral data for CBD classification in order to find the relationship between hyperspectral information and CBDs. Hyperspectral images contain one-dimensional spectral information and two-dimensional spatial information [13]. In food detection modeling based on hyperspectral images, one-dimensional spectral information has often been used for target detection, while two-dimensional spatial information has typically been used for the visualization of the detection results [14]. Currently, one-dimensional spectral information is widely used in prediction modeling about pork tenderness [15], the assessment of *E. coli* contamination [16], and the detection of adulteration in minced mutton [17]. Meanwhile, the results were visualized by applying the model to each pixel in the image. This approach effectively utilizes one- and two-dimensional information to some extent. For the classification of CBDs, it is essential to first determine the location of the defects and then further identify others with similar visual characteristics [18]. This process can begin via the use of two-dimensional spatial information to locate the defects. One-dimensional spectral information is then used to effectively identify the defects of different compositions, allowing more precise identification of the defects within the identified locations.

Whether performing defect localization based on two-dimensional spatial data or defect identification based on one-dimensional spectral data, accurate modeling is required. Convolutional Neural Network (CNN) models excel at precisely extracting positional features from two-dimensional spatial information while also capturing classification features from one-dimensional spectral information [19]. Target detection models based on CNN models (such as Faster R-CNN, YOLO, SSD, etc.) are commonly used for defect detection and grading in food [20]. Studies using YOLOv4 for the defect grading of apples [21], an improved Faster R-CNN for cherry defect identification [22], and SSD for potato defect detection show that CNN models can effectively use two-dimensional spatial information to localize CBDs [23]. Due to the similar visual characteristics of CBDs, it is challenging to achieve accurate identification based solely on two-dimensional spatial information. Therefore, after the CNN models have completed defect localization, extracting one-dimensional spectral information from these localized areas for spectral feature recognition can further improve the accuracy of defect classification. For the one-dimensional spectral information of CBDs, 1-D-CNN can extract the contained classification features through multiple convolutional layers and pooling operations [24], thereby efficiently identifying localized defects [25]. Therefore, based on the CNN model, the basic network frameworks are improved to effectively utilize the multidimensional information obtained from hyperspectral data, which makes it possible to accurately locate and identify CBDs.

**Figure 1:**
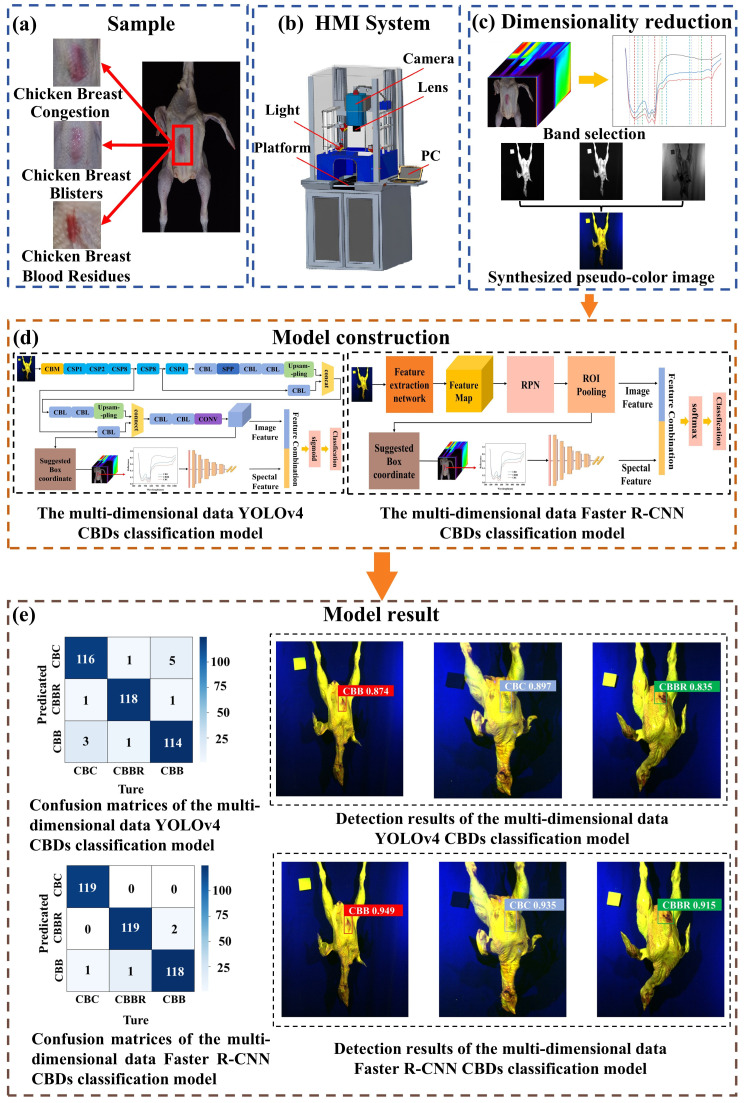
The main flow of the research: (**a**) sample preparation; (**b**) HSI system; (**c**) hyperspectral image dimensionality reduction; (**d**) model construction; (**e**) model result.

The main purpose of this paper is to explore the potential of using hyperspectral imaging technology combined with CNN models in the classification of CBDs. The main workflow of this study is shown in Figure 1. In performing dimensionality reduction and constructing multidimensional data CBD classification models, the aim is to fully utilize the advantages of hyperspectral multidimensional information in identifying substances with similar visual characteristics but different contents in terms of the main components, thereby establishing a method for the localization and identification of CBDs. The specific contents are as follows:

(1) We addressed the redundancy and multidimensional characteristics of hyperspectral data. Using the 1-D CNN CBD classification model, the Improved Firefly Band Selection Algorithm was developed as the objective function of the Firefly Algorithm to obtain the spectral bands most suitable for detecting CBDs.
(2) The YOLOv4 and Faster R-CNN CBD classification models were constructed based on the synthesized pseudo-color images, aiming to evaluate the classification accuracy of CBDs based on two-dimensional spatial data.
(3) The multidimensional YOLOv4 and Faster R-CNN CBD classification models were constructed by introducing the feature extraction module, the 1-D CNN CBD classification model, and the feature fusion layer into the YOLOv4 and Faster R-CNN CBD classification models, respectively. It allows the models to extract classification features from the one-dimensional spectral information of the localized regions and integrate them with the classification features from the two-dimensional spatial information, thereby improving the classification accuracy of CBDs. On this basis, the classification performances of the multidimensional YOLOv4 and Faster R-CNN data CBD classification models were compared.

## 2. Materials and Methods

### 2.1. Sample Preparation

The method proposed in this study is mainly applicable to large slaughterhouses with a high degree of automation, where the production process is generally standardized. This includes procedures such as slaughtering, dehairing, gutting, rinsing, and pre-cooling. The defects generated, the detection environment, and the conveyor system designed for detection in this process are highly similar. Therefore, a representative large slaughterhouse in the local area was selected for sampling in this study. A total of 1080 chicken carcasses (Figure 1a) were selected from different time periods and batches, with a weight range of 1.2 to 2.0 kg and a standard deviation of 0.43 kg. These 1080 chicken carcasses were divided into three groups of 360 each, with each group containing only carcasses of the CBC, CBB, or CBBR types. Water was absorbed from the agitated surface with absorbent paper, and the hyperspectral data of the samples were collected using a hyperspectral imaging system.

### 2.2. Hyperspectral Data Acquisition

The Image-λ-N17E near-infrared HSI system (Sichuan Dualix Spectral Image Technology Co., Ltd., Chengdu, China) (Figure 1b) was used to acquire the hyperspectral data. The system comprised an imaging spectrograph (ImSpector N17E; Spectral Imaging Ltd., Oulu, Finland); an industrial camera (Lumenera lt365R, Lumenera Corporation, Ottawa, ON, Canada) with a sensor resolution of 960 × 1040 pixels and a spectral resolution of 2.8 nm; a camera lens (OLES23; Spectral Imaging Ltd., Oulu, Finland) with an aperture of f/2.4 and a field of view (FOV) of 22o, avoiding imaging errors due to variations in chicken carcass size; two 150 W tungsten filament halogen lamps, providing a continuous and stable light source spanning from the visible to the near-infrared spectral range, covering 400 nm to 2500 nm (Halogen Light source; Sichuan Dua lix Spectral Imaging Technology Co., Ltd., Chengdu, China); a step motor (Isuzu Optics Corp., Taiwan, China)-driven displacement platform; and a computer equipped with data acquisition software.

Hyperspectral data acquisition was carried out in the factory workshop, and the hyperspectral camera was used to scan and collect data, employing a line camera in conjunction with a displacement platform. The acquisition parameters were set as follows: an object distance of 40 cm; a platform forward speed of 0.6 cm/s; a backward speed of 2 cm/s; an exposure time of 10 ms. The hypercube comprised 360 single-band spectral images, each 960 pixels in width and 1101 pixels in length, spanning the wavelength range from 382.3 nm to 1020.2 nm with a spectral resolution of 1.8 nm. During data acquisition, in order to reduce the impact of surface unevenness on the quality of the hyperspectral images, two halogen lamps were used to uniformly illuminate the surface of the chicken carcass in opposing directions, ensuring an even distribution of reflected light. Additionally, the chicken carcass was kept at the center of the camera’s field of view to avoid angular deviations that could cause inconsistency in imaging, ensuring that each image accurately and completely reflected the characteristics of the chicken carcass.

### 2.3. Spectral Image Correction

Due to unstable light sources, camera currents, and other factors, noise may be introduced during hyperspectral data acquisition, which will impact the imaging quality. To mitigate external noise interference, black and white correction was utilized at the pixel level for hyperspectral image correction. Specifically, the black calibration image is obtained by turning off lights and covering the camera lens, while the white calibration image is captured by placing a white cloth next to the chicken carcass. The specific correction calculation formula is shown in Equation (1):

(1) $$R=\frac{\left(I-B\right)}{\left(W-B\right)}\times 100\%$$

where *I* denotes the original image; *W* denotes the gray and white calibration image; *B* denotes the all-black calibration image; and *R* denotes the corrected relative image.

### 2.4. Hyperspectral Band Selection Algorithm

**Figure 2:**
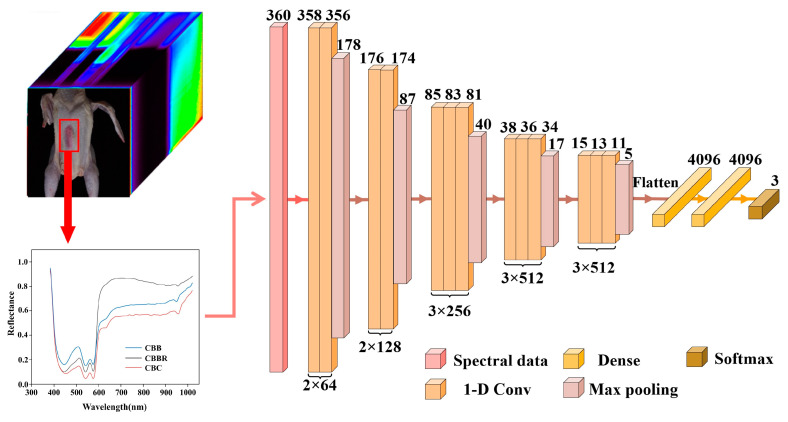
The structure of the 1-D CNN CBD classification model.

Extracting effective bands from the vast amount of hyperspectral data in order to classify CBDs is crucial for improving the classification accuracy and model detection speed. The Firefly Algorithm was chosen as the fundamental dimensionality reduction algorithm to be used for hyperspectral data in this paper. The algorithm has excellent global search capabilities, and can avoid local optima due to the large volume of data processed during dimensionality reduction, thus affecting the accuracy of band selection. It was proposed by Yang in 2008 and simulates the process of light signal exchange between fireflies [26]. The flow of the algorithm is as follows: firstly, the population is initialized and the luminance value of each firefly is calculated; then, based on the luminance difference and distance, the firefly with lower luminance values moves closer to the one with higher luminance values; then, the position is updated based on the luminance attractiveness and the luminance is recalculated iteratively until the stopping condition is satisfied, thus finding the global optimal solution. Only accurate evaluation results can guide the algorithm towards better solutions in iterative processes. Therefore, an accurate evaluative object function is crucial for the firefly classification in terms of performance. In this paper, the 1-D CNN CBD classification model was constructed based on the characteristics of one-dimensional spectral data, serving as the objective function of the Firefly Algorithm. The structure of the 1-D CNN CBD classification model is shown in Figure 2. It consists of 13 one-dimensional convolutional layers (1-D Conv) with ReLU activation functions, 5 max pooling layers, and 2 fully connected layers. Using the 1-D CNN CBD classification model as the objective function, the improved Firefly Band Selection Algorithm was constructed. The initial parameters were set as follows: number of fireflies (n) = 5, where each firefly represents three different hyperspectral bands; maximum attraction ($\beta_{0}$) = 0.5; light intensity absorption coefficient (λ) = 0.8; step coefficient (∂) = 0.6; and maximum number of iterations (Max Iterations) = 100. The 360-band average spectral reflectance data for CBDs were extracted from the hyperspectral data using ENVI 5.6 software (Harris Geospatial Solutions, Interlocken, CO, USA) and used as the Improved Firefly Band Selection Algorithm input.

### 2.5. The Multidimensional Data CBD Classification Model

**Figure 3:**
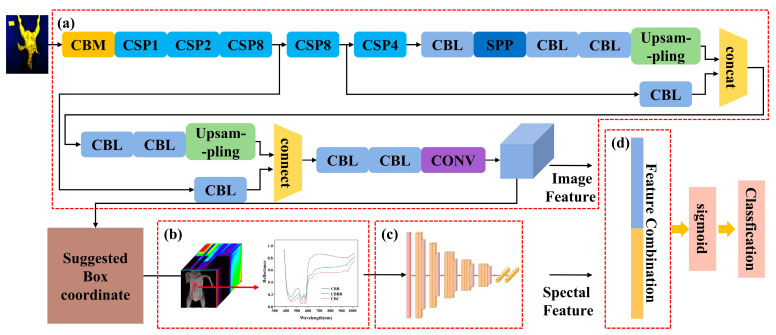
The structure of the multidimensional data YOLOv4 CBD classification model: (**a**) improved head part; (**b**) the spectral data extraction module; (**c**) the 1-D CNN CBD classification model for one-dimensional feature extraction; (**d**) the feature fusion layer.

For the classification of CBDs, based on the advantages of hyperspectral two-dimensional images in terms of location information and the advantages one-dimensional spectral information in terms of composition and ingredients, two-dimensional images were used to locate the defects. Then, one-dimensional spectral data were extracted from the defect areas for identification in this paper. To accurately identify CBDs, it is essential to construct a CNN model that effectively utilizes both one-dimensional and two-dimensional information, and then integrates this information. YOLOv4 and Faster R-CNN are representative models among CNN models, being capable of both localizing defects and identifying defects within the localized regions [27]. However, YOLOv4 and Faster R-CNN primarily classify based on two-dimensional image features. Due to the similar visual characteristics of CBDs, performing effective identification based solely on two-dimensional image information is challenging. To improve the identification accuracy of the model for CBDs, the classification features from two-dimensional spatial data need to be fused with those from one-dimensional spectral data. In view of this, multidimensional data classification models for CBDs have been developed based on YOLOv4 and Faster R-CNN, respectively. Taking the network structure of YOLOv4 as an example, the original YOLOv4 structure was modified, with the spectral data extraction module (Figure 3b), the 1-D CNN CBD classification model for one-dimensional feature extraction (Figure 3c), and the feature fusion layer (Figure 3d) added to YOLOv4. As shown in Figure 3, the multidimensional data YOLOv4 CBD classification model was constructed. Figure 3a details the structure of the head parts of the modified YOLOv4 network. The head part of YOLOv4 network performs the prediction of localized CBDs, which includes three feature extraction branches, each handling feature maps of different scales to enhance detection capabilities for defects of various sizes. To simplify the network structure while maintaining the integrity of the feature extraction for CBDs, only the largest feature extraction branch of the head part (Figure 3a) was retained in the modified model.

**Figure 4:**
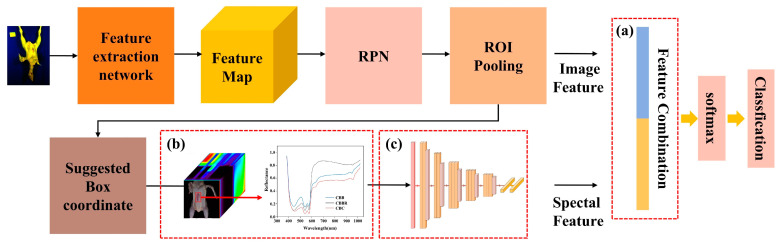
The structure of the Faster R-CNN CBD classification model, which uses multidimensional data: (**a**) the feature fusion layer; (**b**) the spectral data extraction module; (**c**) the 1-D CNN CBD classification model for one-dimensional feature extraction.

Faster R-CNN, like YOLOv4, relies on two-dimensional image features for classification. Faster R-CNN uses two-dimensional convolution, max pooling, and fully connected layers to achieve the feature extraction, localization, and identification of CBDs, respectively. Among these features, the Region Proposal Network (RPN) in Faster R-CNN has two decision branches: one is responsible for determining whether the generated anchors contain CBDs, and the other is responsible for obtaining coordinate adjustment parameters. At the end of the RPN, initial rough detection results are obtained. The CBD classification based on Faster R-CNN has the same limitations as the use of YOLOv4. Similarly, to improve the identification accuracy of defects and to compare the accuracy of these two models in multidimensional data identification, the spectral data extraction module (Figure 4a), the 1-D CNN CBD classification model for one-dimensional feature extraction (Figure 4b), and the feature fusion layer (Figure 4c) were also added to the structure of Faster R-CNN. The multidimensional data Faster R-CNN CBD classification mode, shown in Figure 4, was constructed.

### 2.6. Evaluation Indicators

To evaluate and compare the two models more objectively, mIoU, precision, recall, F1 score, AP, mAP, and inference time were used as the evaluation indexes of the multidimensional data localization and identification model, and the confidence level in terms of precision, recall, and F1 score was set to 0.5. IoU (intersection over union) is a metric used to evaluate the overlap between the predicted bounding box, generated by the model, and the ground truth bounding box during data annotation, while mIoU (mean intersection over union) is the average IoU across multiple images, and is used to assess the overall localization accuracy of the model. Precision represents the proportion of true positive samples in relation to predicted positive samples: the higher the precision value, the higher prediction accuracy of the model. Recall is an indicator of the model’s ability to identify all positive samples; the higher the recall, the lower the model’s miss rate [28]. F1 score is the PR area enclosed by the curve (precision is the vertical coordinate and recall is the horizontal coordinate) combining precision and recall. The higher the F1 score at the same confidence level, the better the performance of the model. AP and mAP are the indices generated within a certain threshold. They not only evaluate the results, but also reflect the stability of the model for each classification prediction. The smaller the fluctuation of the PR curve and the larger the value of AP, the better the stability and accuracy of the model. Inference time represents the time required for a model to process a single image and is used as a metric to evaluate the model’s speed of detection. When the detection accuracy meets the requirements, a shorter inference time indicates the better real-time performance of the model. The corresponding calculation results are shown in Equations (2)–(7):

(2) $$\mathrm{Io}U=\frac{\left|A\cap B\right|}{\left|A\cup B\right|}$$

(3) $$Accuracy=\frac{\left(TP+TN\right)}{\left(P+N\right)}$$

(4) $$Precision=\frac{TP}{\left(TP+FP\right)}$$

(5) $$Recall=\frac{TP}{\left(TP+FN\right)}$$

(6) $$AP=\int_{0}^{1}Precision\;d\left(Recall\right)$$

(7) $$mAP=\frac{AP}{C}$$

where *A* is the predicted region and *B* is the real region. *TP* (true positive) is the number of samples that are correctly predicted by the model to belong to a category and do belong to a category, *TN* (true negative) is the number of samples that do not belong to a category and are not predicted by the model to belong to a category, *FP* (false positive) is the number of samples that are incorrectly predicted by the model to belong to a category and are not, *C* is the number of categories, and *AP* is the area bounded by the PR curve and the horizontal and vertical coordinates (with recall as the horizontal coordinate and precision as the vertical coordinate).

## 3. Results

### 3.1. Spectral Characteristics

**Figure 5:**
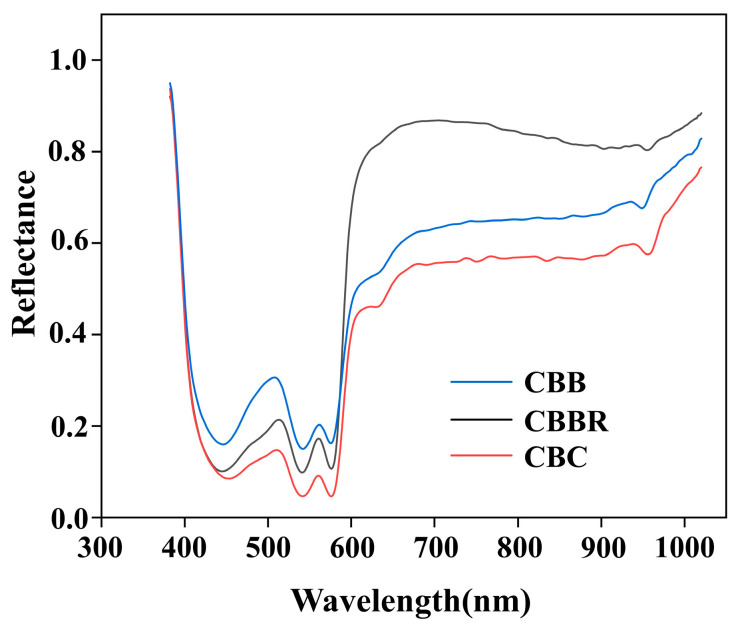
Mean spectral reflectance curves of CBDs.

Due to the different moisture, protein, and other contents in various CBDs, their spectral performances also differ. As shown in Figure 5, by calculating the average spectral reflectance of breast defects (CBC, CBBs, and CBBRs) in each hyperspectral image, the average spectral curves of all CBDs were obtained. It can be seen that the three defect types have distinct absorption peaks in the 420–600 nm wavelength range and a smaller absorption trough in the 950–970 nm range. The wavelength ranges of 420–450 nm, 520–570 nm, and 570–600 nm represent proteins that correspond to the main hemoglobin components of the blood [29]. These values are consistent with the three defects being closely related to blood composition. Due to the different causes of defect formation, the number of red blood cells and the hemoglobin content of the above three blood-related defects are different. CBC results from internal bleeding due to a chest impact, CBB results from capillary rupture and bleeding due to prolonged friction, and CBBR is blood that was not completely rinsed off the surface after external bleeding. Therefore, the hemoglobin content decreases sequentially from CBC to CBBRs to CBBs. This is reflected in the spectral curves as a sequential decrease in absorption intensity in the protein absorption bands of CBC, CBBs, and CBBRs. The band near 960 nm is related to water absorption [30]. CBBs contain tissue fluid, and CBC contains plasma, and both of these are primarily composed of water. CBBRs, having been air-dried, have the least amount of moisture compared to the other two defects. This is reflected in the spectral curves near 960 nm, where the spectral reflectance decreases sequentially from CBC to CBBs to CBBRs. The trends and differences in the spectral curves indicate that it is feasible to classify CBC, CBBs, and CBBRs using hyperspectral data.

### 3.2. Band Selection Results and Analysis

#### 3.2.1. The Training of the Objective Function

**Figure 6:**
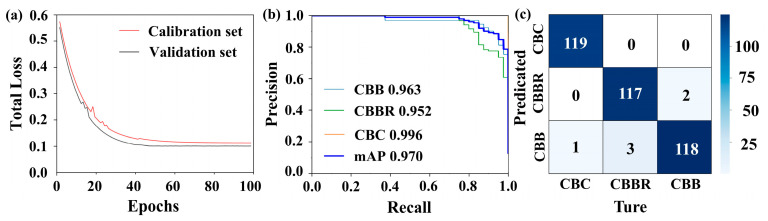
Results of the 1-D CNN CBD classification model: (**a**) loss curves; (**b**) PR curves; (**c**) confusion matrices.

The presence of redundant or irrelevant information in hyperspectral data can lead to model overfitting, while dimensionality reduction can lead to key features being retained, thereby improving the model’s generalization ability. In this paper, the Firefly Algorithm is chosen as the dimensionality reduction method for hyperspectral images. For hyperspectral data dimensionality reduction using the Firefly Algorithm, an objective function capable of accurately evaluating the classification performance of each band combination represented by each firefly in every iteration is necessary. In this paper, the 1-D CNN CBD classification model, based on one-dimensional spectral information, was established and used as the objective function of the Firefly Algorithm. The training results of the 1-D CNN CBD classification model for over 100 training cycles are shown in Figure 6. It illustrates that the model starts to converge in the 35th generation and that the loss values for the calibration and validation sets are 0.12 and 0.1, respectively, which indicates that the 1-D CNN has strong adaptability to the spectral data of CBDs, making it suitable for the identification of these defects (Figure 6a). Additionally, the evaluation metrics in Table 1 show that the F1 scores for each class are all above 0.95, confirming the model’s good performance. The AP values also exceed 0.95, indicating the model’s good stability. However, in terms of individual categories, examining the confusion matrix in Figure 6c, we can see that there is some degree of confusion in the classification of CBBs and CBBRs, which is also evidenced by the relatively small fluctuations in the PR curve in Figure 6b [31]. This may be due to the small differences in erythrocyte count between these two defects and the fact that some CBBC surfaces were not fully air-dried, leading to spectral reflectance values that are too close. This makes it difficult for the model to discriminate. The above indicators suggest that the 1-D CNN CBD classification model is suitable for use as the objective function for the Firefly Algorithm and as a method for one-dimensional spectral feature extraction in subsequent classification models. However, some confusion was observed in the classification of CBBs and CBBRs by the model, indicating that the classification of CBDs based on a single dimension has certain limitations.

#### 3.2.2. Band Selection Result

**Figure 7:**
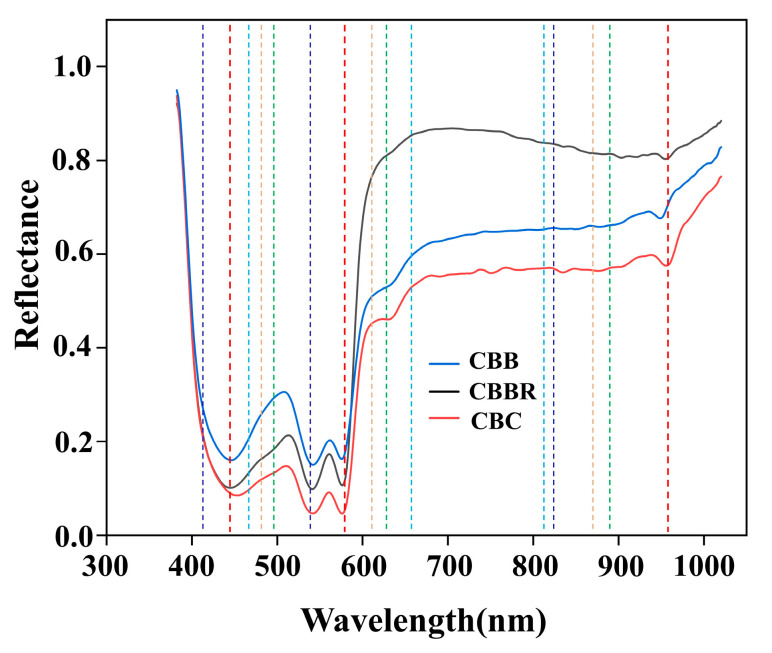
Results of band selection.

**Figure 8:**
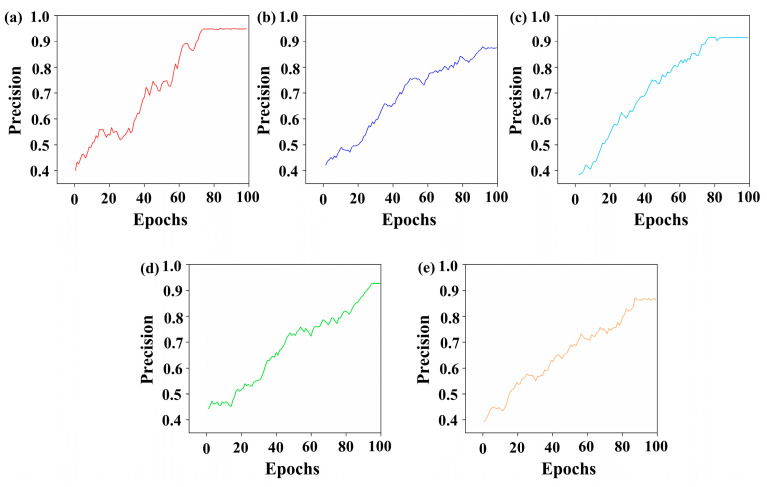
The variation in the defect recognition accuracy of the 5 band combinations selected by the Improved Firefly Band Selection Algorithm: (**a**–**e**) are the variations in defect identification accuracy for each band combination. The corresponding band combinations are 430 nm, 576 nm, and 962 nm; 410 nm, 525 nm, and 815 nm; 457 nm, 662 nm, and 809 nm; 498 nm, 621 nm, and 895 nm; and 489 nm, 605 nm, and 872 nm, respectively.

**Figure 9:**
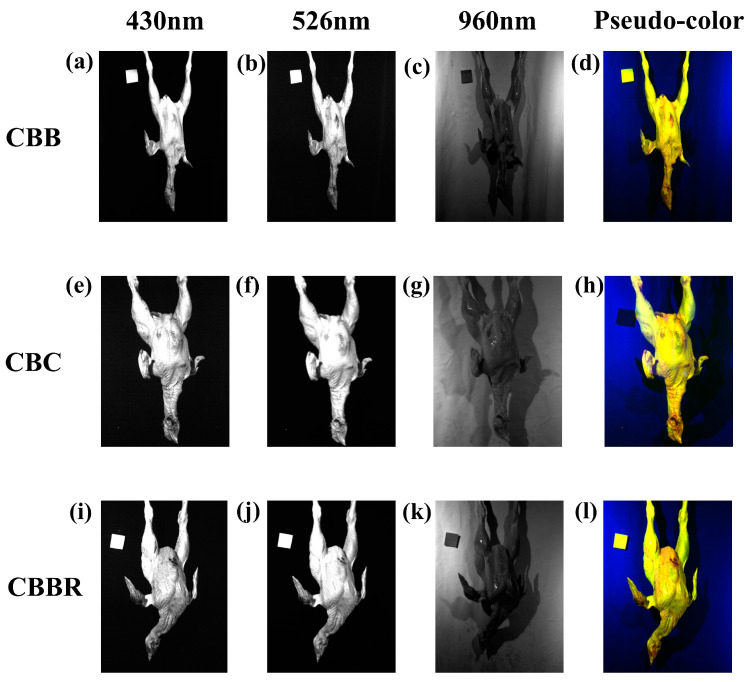
Pseudo-color images synthesized using grayscale images. The grayscale images of CBBs (**a**–**c**), CBC (**e**–**g**), and CBBRs (**i**–**k**) at 430 nm, 576 nm, and 962 nm are synthesized into pseudo-color images (**d**,**h**,**l**).

The results of the Improved Firefly Band Selection Algorithm after 100 rounds of band selection are shown in Figure 7. The dashed lines represent the five different band combinations associated with the five fireflies set by the Hyperspectral Band Selection Algorithm, with each color representing one combination. Each combination includes three bands, facilitating subsequent modeling based on the band selection results. The five selected band combinations are discretely distributed across the entire wavelength range, indicating that the algorithm performed a global search over the hyperspectral wavelength range and avoided local optima. Figure 8a–e show the changes in CBD classification efficiency of the 5 band combinations selected by the Improved Firefly Band Selection Algorithm after 100 iterations. In Figure 8, the classification efficiencies of the band combinations represented by each firefly are 0.96, 0.83, 0.91, 0.89, and 0.90, respectively. Among these, the red band combination has the highest classification efficiency of 0.96 (Figure 8a), indicating that this band combination is the optimal choice for identifying CBDs. Therefore, the grayscale images corresponding to the band combination represented by the red dashed line (430 nm, 576 nm, and 962 nm) are synthesized into pseudo-color images and used as the input in terms of two-dimensional spatial information in subsequent models. Figure 9a–c and Figure 9d, respectively, show the grayscale and pseudo-color images of CBC at wavelengths of 445 nm, 576 nm, and 962 nm, while Figure 9e–g, and Figure 9h, as well as Figure 9i–k, and Figure 9l, respectively, show the grayscale and pseudo-color images of CBBs and CBBRs at the same wavelengths. In the pseudo-color images constructed from the downscaled images, the color differences between the three types of defects and the surrounding skin were preserved, while the differences between the defects were significantly enhanced. This provides a better image environment for CBD identification, based on the pseudo-color image breast defects, and the classification of CBDs later on.

### 3.3. Results of the CBD Classification Model

#### 3.3.1. Results of the CBD Classification Model Based on Pseudo-Color Images

**Figure 10:**
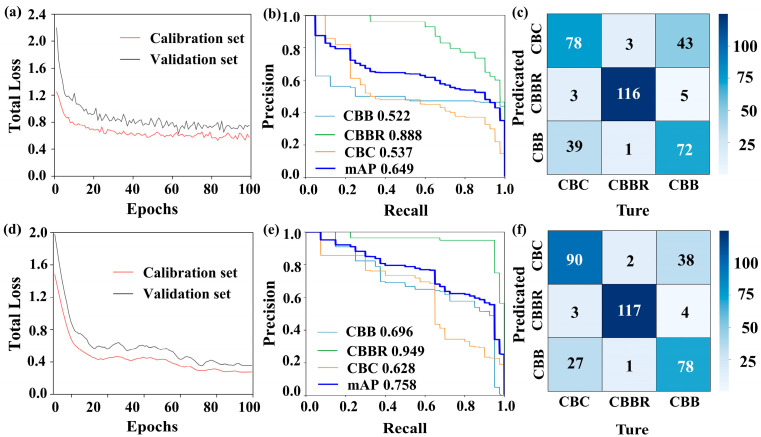
The specific training results of the CBD classification model based on pseudo-color images: (**a**–**c**) are the loss curves, PR curves, and confusion matrices of the YOLOv4 CBD classification model; (**d**–**f**) are the loss curves, PR curves, and confusion matrices of the Faster R-CNN CBD classification model.

**Figure 11:**
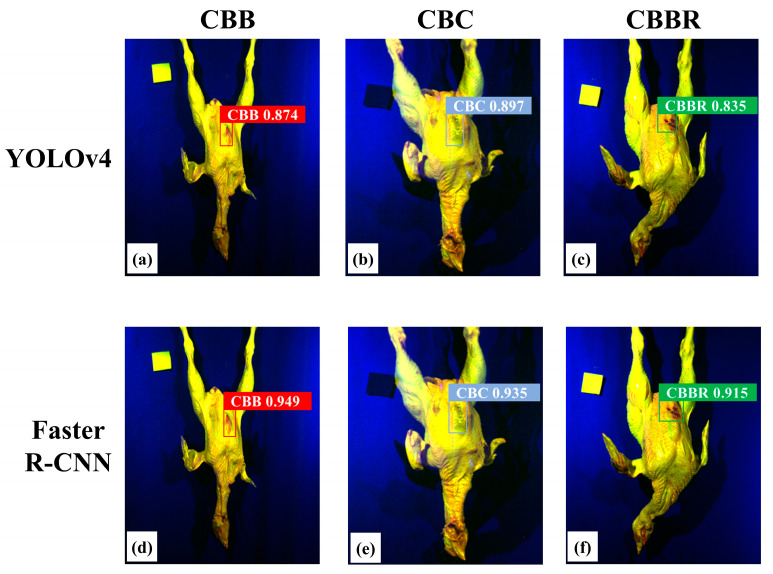
Randomly selected detection results of the model: (**a**–**c**) and (**d**–**f**) are the random detection results of the YOLOv4 CBD classification model and the Faster R-CNN CBD classification model, respectively, for chicken carcasses with CBB, CBC, and CBBR defects.

First, we investigated the efficiency of YOLOv4 and Faster R-CNN in localizing and identifying CBDs based solely on two-dimensional spatial information. In this paper, synthesized pseudo-color images (Figure 9) were used to construct YOLOv4 and Faster R-CNN CBD classification models. Figure 10 shows the specific training results of the YOLOv4 and Faster R-CNN CBD classification models when used to classify CBDs. The convergence process of the LOSS values for both models during training is shown in Figure 10a,d. The LOSS value of the YOLOv4 CBD classification model rapidly decreases within the first 20 epochs, gradually stabilizing and converging after 80 epochs. In contrast, the LOSS value of the Faster R-CNN CBD classification model steadily decreases within the first 30 epochs, but then slowly stabilizes and converges after 70 epochs. This indicates that applying the pseudo-color image data set, synthesized from grayscale images selected by The Improved Firefly Band Selection Algorithm, to the YOLOv4 and Faster R-CNN CBD classification models is feasible. The localization and identification results of the CBD classification model for randomly selected samples are shown in Figure 11, where differently colored boxes represent different defects, with predicted labels and IoU values displayed above the boxes. Figure 11 shows that both models are able to localize different CBDs. The mIoU values of the two models are 0.903 and 0.932, and the IoU values for different CBDs do not show significant differences (Table 2). This shows that both models perform the defect localization task effectively, with the Faster R-CNN CBD classification models showing higher localization accuracy. However, as can been seen from the confusion matrices (Figure 10c,f), the defect identification performance of both models is not ideal, particularly for YOLOv4. The mAP value is 0.649. It is slightly higher for Faster R-CNN, where it is 0.758 (Table 2). Additionally, compared with CBBR identification, both models exhibit more severe confusion when identifying CBC and CBBs, likely due to their similar compositions. Both are subcutaneous hemorrhages in chicken breasts, displaying similar morphologies and structures, but CBBs contain more tissue fluid, increasing the difficulty of classification based on two-dimensional images. The similar trends in the PR curves for these two defects, shown in Figure 10b,e, further confirm that these two defects have similar visual characteristics. CBBR, which is blood residue on the surface of the chicken breast, differs from CBC and CBBs in terms of shape and color. Therefore, the identification performance for CBBRs is relatively better than that for these two defects. As shown in Table 2, the inference time of the YOLOv4 and Faster R-CNN CBD classification models reaches a maximum of 35.9 ms, demonstrating that CBD detection based on pseudo-color images has good real-time performance. The mIoU values of both models exceed 0.903, indicating that they can accurately localize CBDs. However, the lowest mAP and F1 scores for the two models are 0.522 and 0.621, respectively. This is mainly due to the significant similarities in image features among the three types of defects, particularly between CBC and CBBs. This highlights the limitations of the current models in identifying CBDs based solely on two-dimensional spatial information. Therefore, it is necessary to incorporate information from other dimensions to improve classification performance.

#### 3.3.2. Results of the Multidimensional Data CBD Classification Model

Using the characteristics of the multidimensional information obtained from hyperspectral spectra, the above synthetic pseudo-color images (Figure 10) and one-dimensional spectral information about CBDs were used as inputs to construct the multidimensional data CBD classification model. The network structures of YOLOv4 and Faster R-CNN were modified based on the morphology of CBDs and the characteristics of hyperspectral data, and multidimensional data CBD classification models were constructed. With modified models based on the realization of defect localization, the spectral values of the defects in the localized region are extracted, and the defect images and spectral information are fused to achieve the accurate identification of CBDs. The structures of the multidimensional data YOLOv4 CBD classification model and the multidimensional data Faster R-CNN CBD classification model are shown in Figure 3 and Figure 4. These models were constructed by incorporating the spectral data extraction module, the 1-D CNN CBD classification model, and the feature fusion layer into the original YOLOv4 and Faster R-CNN frameworks and by removing redundant network structures in YOLOv4.

**Figure 12:**
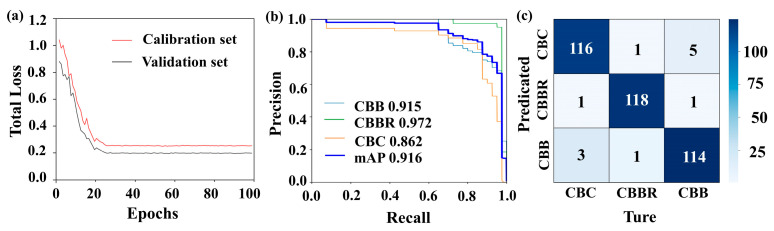
Detection results of the multidimensional data YOLOv4 localization and identification model: (**a**) loss curves; (**b**) PR curves; (**c**) confusion matrices.

Figure 12 shows the detection results of the multidimensional data YOLOv4 CBD classification model when applied to the test set. From Figure 12a, it can be observed that the loss values of the model on the training and test sets converge after 100 iterations, reaching 0.224 and 0.208, respectively. Figure 12b presents the PR curve of the model, showing that the classification accuracy for CBDs exceeds 0.862. These results indicate that the YOLOv4 CBD classification model based on multidimensional data achieved a good fit on the multidimensional data, and its classification accuracy for CBDs improved to varying degrees compared to the YOLOv4 model based on pseudo-color images. The confusion matrix shown in Figure 12c reflects the classification confusion between different defect types. Compared to the original YOLOv4, the model shows a reduced degree of classification confusion for various types of CBD. However, there is still some confusion between CBC and CBBs. This confusion may be due to the fact that both CBC and CBBs are subcutaneous hemorrhages in the breast, with CBBs containing more tissue fluid, leading to very similar visual characteristics and spectral information, which increases the difficulty of classification. Additionally, the relatively low localization accuracy of YOLOv4 leads to there being more noise in the extracted spectral information, further impacting the classification of CBC and CBBs. Table 3 provides a more comprehensive and detailed evaluation of the model. It can be seen from Table 3 that the model’s IoU values remain similar to those of the original YOLOv4 model, with F1 scores and AP values for all defect types above 0.862, while the detection time increases by only 12.1 ms. This indicates that the introduction of one-dimensional spectral information did not affect the model’s localization accuracy, and that using the multidimensional data fusion method for CBD classification significantly improved the model’s classification accuracy with only a minimal increase in time cost.

**Figure 13:**
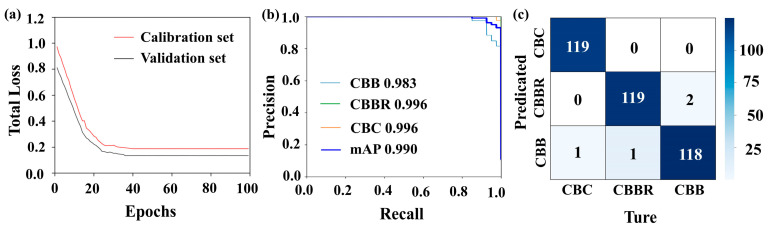
Detection results of the multidimensional data Faster R-CNN CBD classification model: (**a**) loss curves; (**b**) PR curves; (**c**) confusion matrices.

Figure 13 shows the detection results of the multidimensional data Faster R-CNN CBD classification model when applied to the test set. The multidimensional data Faster R-CNN CBD classification model has loss values of 0.003 and 0.015 when applied the training and test sets, respectively, with classification accuracies exceeding 98% (as shown in Figure 13a,b). This indicates that, compared to the multidimensional data YOLOv4 CBD classification model, the multidimensional data Faster R-CNN CBD classification model demonstrates improved fitting performances and classification accuracies when applied to multidimensional data. From the confusion matrix shown in Figure 13c, it is evident that the multidimensional data Faster R-CNN CBD classification model further reduces the classification confusion for CBC and CBBs, which poses a challenge for the multidimensional data YOLOv4 CBD classification model. This improvement is due to the Faster R-CNN’s feature extraction network, which can capture the deeper classification features of CBC and CBBs from pseudo-color images. Additionally, Faster R-CNN can more precisely localize the defects, reducing noise in the one-dimensional spectral information and thereby enhancing classification accuracy. Table 4 provides a more comprehensive and detailed evaluation of the models. As shown in Table 4, the multidimensional data Faster R-CNN CBD classification model outperformed the multidimensional data YOLOv4 CBD classification model in both localization accuracy (IoU) and AP and F1 scores, and the detection time increased to 58.2 ms.

**Figure 14:**
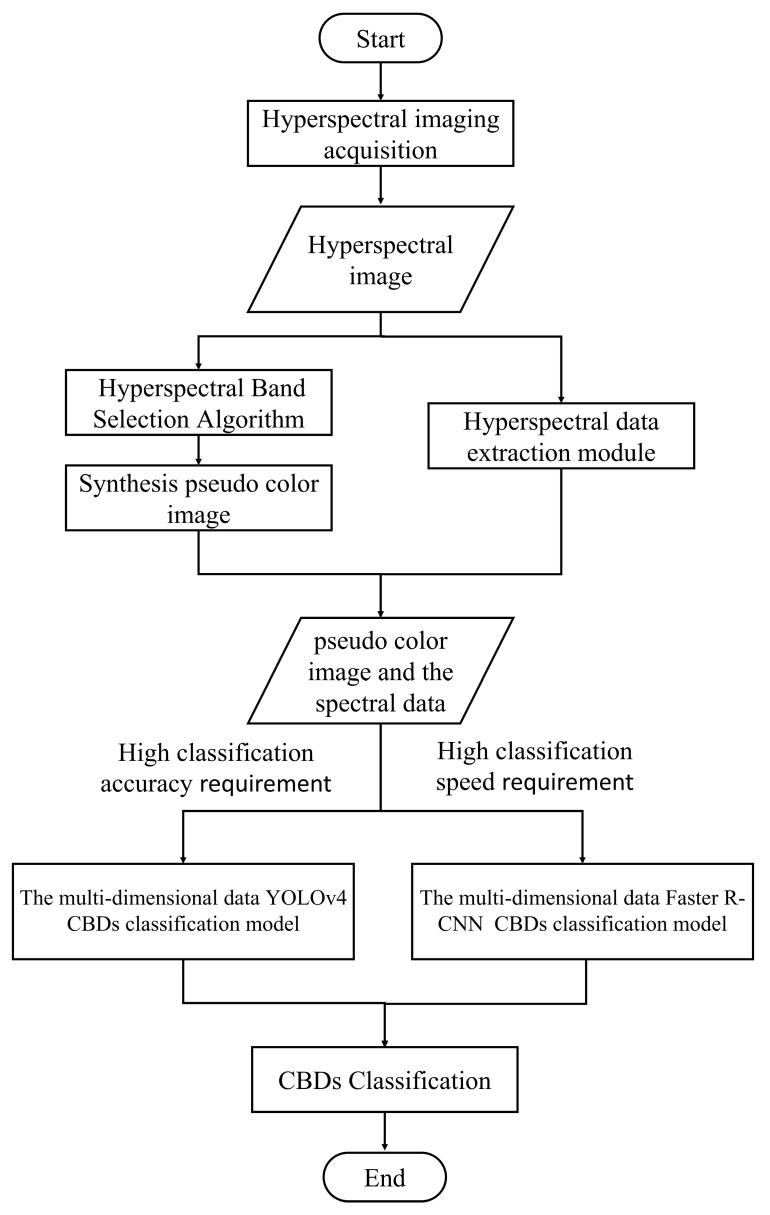
The main task of the multidimensional data classification model is the classification of CBDs.

In summary, multidimensional data classification models based on hyperspectral data are capable of performing the classification tasks for CBDs. The main detection flow of the model is shown in Figure 14. However, different model architectures have different focuses in terms of classification: the multidimensional data Faster R-CNN localization and identification model exhibits higher classification and localization accuracy, while the multidimensional data YOLOv4 localization and identification model provides faster classification speed. In practical production, different models can be selected based on specific needs in terms of classifying CBDs. It should be emphasized that, as the classification accuracy of the model will be affected by the field environment and chicken species, when in a new detection environment, more hyperspectral images of chicken carcasses in the current environment need to be collected to correct and add training to the model in order to maintain the original classification accuracy.

## 4. Conclusions

In this paper, a method for CBD classification was proposed based on hyperspectral images and CNN models. The 1-D CNN CBD classification model was chosen as the objective function for the Firefly Algorithm in order to construct the Improved Firefly Algorithm, which aimed to enhance the accuracy of band selection. As a result, 445 nm, 576 nm, and 962 nm were selected as the most suitable bands for CBD classification. Based on the pseudo-color images synthesized from the dimensionally reduced hyperspectral data and the Faster R-CNN and YOLOv4 network models, a CBD classification model was developed. The classification model based on pseudo-color images performed well in defect localization, with mIoU values of 0.903 and 0.932. However, its performance in CBD classification was not ideal, with mAP values of 0.649 and 0.705. To improve the classification accuracy based on two-dimensional spatial information, a multidimensional data CBD classification model was constructed. The multidimensional data CBD classification model successfully classifies CBDs, but the focus of the classification varies across different model architectures. The multidimensional data YOLOv4 CBD classification model achieved an mAP of 0.970 with an inference time of 41.8 ms, while the multidimensional data Faster R-CNN CBD classification model, although having a longer inference time of 58.2 ms, achieved a higher mAP of 0.990. Currently, there is limited international research on localizing and identifying CBDs. This paper proposes a model using hyperspectral data for the automatic and rapid localization and identification of CBDs and tests the efficiency of two CNN models. This could provide valuable insights for the automation of broader livestock and poultry detection.

## Figures and Tables

**Table 1:** A comparison of the 1-D CNN CBD classification models’ performances in CBD classification.

Label	Precision	Recall	F1 Score	AP	mAP
CBC	0.992	0.991	0.992	0.996	0.970
CBB	0.977	0.984	0.980	0.963	
CBBR	0.976	0.969	0.972	0.952	

**Table 2:** Performance metrics comparison of the localization and identification model based on pseudo-color images.

Model	Label	Precision	Recall	F1 Score	AP	IoU	mIoU	mAP	Inference Time
YOLOv4	CBC	0.634	0.613	0.624	0.522	0.894	0.903	0.649	29.7
	CBB	0.600	0.643	0.621	0.537	0.909			
	CBBR	0.967	0.935	0.951	0.888	0.902			
Faster R-CNN	CBC	0.750	0.692	0.720	0.628	0.947	0.932	0.758	35.9
	CBB	0.787	0.783	0.808	0.696	0.925			
	CBBR	0.975	0.944	0.959	0.949	0.934			

**Table 3:** A comparison of the multidimensional data YOLOv4 CBD classification models.

Label	Precision	Recall	F1 Score	AP	IoU	mIoU	mAP	Inference Time
CBC	0.967	0.951	0.959	0.915	0.894	0.901	0.916	41.8
CBB	0.950	0.966	0.958	0.862	0.912			
CBBR	0.983	0.983	0.983	0.972	0.897			

**Table 4:** A comparison of the multidimensional data Faster R-CNN localization and identification model’s performance on different carcass breast blood-related defects.

Label	Precision	Recall	F1 Score	AP	IoU	mIoU	mAP	Inference Time
CBC	0.992	1.000	0.996	0.996	0.919	0.924	0.990	58.2
CBB	0.983	0.992	0.983	0.983	0.924			
CBBR	0.992	0.983	0.979	0.988	0.935			

## Data Availability

The original contributions presented in this study are included in the article. Further inquiries can be directed to the corresponding author.

## References

[1] Uzundumlu A.S., Dilli M. (2023). Estimating Chicken Meat Productions of Leader Countries for 2019–2025 Years. Cienc. Rural.

[2] Beski S.S.M., Swick R.A., Iji P.A. (2015). Specialized protein products in broiler chicken nutrition: A review. Anim. Nutr..

[3] Aslan R., Sarıca M., Çavdarcı H., Erensoy K., Karaçay N. (2024). The use of partially slatted floor designs as an alternative to littered systems in broiler chickens. I. The effects on the performance, slaughter and carcass traits. Trop. Anim. Health Prod..

[4] Shi J., Sun G., Tian Y., Han R., Li G., Huang Y., Wang J., Kang X. (2012). Screening genes related to breast blister (keel cyst) in chicken by delta differential display. Asian J. Anim. Vet. Adv..

[5] Ahn J.-H., Jang W.-J., Lee W.-H., Kim J.-D. (2020). Detection of needles in meat using X-ray images and convolution neural networks. J. Sens. Sci. Technol..

[6] Lu B., Dao P.D., Liu J., He Y., Shang J. (2020). Recent advances of hyperspectral imaging technology and applications in agriculture. Remote Sens..

[7] Ma J., Sun D.W., Pu H., Cheng J.H., Wei Q. (2019). Advanced Techniques for Hyperspectral Imaging in the Food Industry: Principles and Recent Applications. Annu. Rev. Food Sci. Technol..

[8] Kang R., Yang K., Zhang X.X., Wu W., Chen K.J. (2016). Development of online detection and processing system for contaminants on chicken carcass surface. Appl. Eng. Agric..

[9] Taemin K., Byoung-Kwan C., Moon K. (2010). Emission filter design to detect poultry skin tumors using fluorescence hyperspectral imaging. Rev. Colomb. Cienc. Pecu..

[10] Ahmadi S.A., Mehrshad N., Razavi S.M. (2018). Semisupervised dimensionality reduction for hyperspectral images based on the combination of semisupervised learning and metric learning. Imaging Sci. J..

[11] Sawant S., Manoharan P. (2020). Hyperspectral band selection based on metaheuristic optimization approach. Infrared Phys. Technol..

[12] Su H., Yong B., Du Q. (2015). Hyperspectral band selection using improved firefly algorithm. IEEE Geosci. Remote Sens. Lett..

[13] Zhu M., Huang D., Hu X.J., Tong W.H., Han B.L., Tian J.P., Luo H.B. (2020). Application of hyperspectral technology in detection of agricultural products and food: A Review. Food Sci. Nutr..

[14] Caporaso N., ElMasry G., Gou P. (2021). Chapter 13—Hyperspectral imaging techniques for noncontact sensing of food quality. Innovative Food Analysis.

[15] Tao F., Peng Y., Li Y., Chao K., Dhakal S. (2012). Simultaneous determination of tenderness and Escherichia coli contamination of pork using hyperspectral scattering technique. Meat Sci..

[16] Li D., Zhang F., Yu J., Chen X., Liu B., Meng X. (2021). A rapid and non-destructive detection of Escherichia coli on the surface of fresh-cut potato slices and application using hyperspectral imaging. Postharvest Biol. Technol..

[17] Kamruzzaman M., Sun D.W., ElMasry G., Allen P. (2013). Fast detection and visualization of minced lamb meat adulteration using NIR hyperspectral imaging and multivariate image analysis. Talanta.

[18] Xueying L., Zongmin L., Huimin Q., Guangyuan C., Pingping F. (2023). Soil carbon content prediction using multi-source data feature fusion of deep learning based on spectral and hyperspectral images. Chemosphere.

[19] Jernelv I.L., Hjelme D.R., Matsuura Y., Aksnes A. (2020). Convolutional neural networks for classification and regression analysis of one-dimensional spectral data. arXiv.

[20] Liu Y., Pu H., Sun D.-W. (2021). Efficient extraction of deep image features using convolutional neural network (CNN) for applications in detecting and analysing complex food matrices. Trends Food Sci. Technol..

[21] Liang X., Jia X., Huang W., He X., Li L., Fan S., Li J., Zhao C., Zhang C. (2022). Real-Time Grading of Defect Apples Using Semantic Segmentation Combination with a Pruned YOLO V4 Network. Foods.

[22] Wei R., Pei Y., Jiang Y., Zhou P., Zhang Y. (2021). Detection of cherry defects based on improved Faster R-CNN model. Food Mach..

[23] Wang C., Xiao Z. (2021). Potato surface defect detection based on deep transfer learning. Agriculture.

[24] Yang B., Yang Z., Xu Y., Cheng W., Zhong F., Ye D., Weng H. (2024). A 1D-CNN model for the early detection of citrus Huanglongbing disease in the sieve plate of phloem tissue using micro-FTIR. Chemom. Intell. Lab. Syst..

[25] Shaoxiong Y., Guangman S., Guangqing H., Quan W. (2022). Reshaping Hyperspectral Data into a Two-Dimensional Image for a CNN Model to Classify Plant Species from Reflectance. Remote Sens..

[26] Kumar G.V.S., Leelawathee S., Keerthana V., Thushitha S. (2017). A Novel Approach for Band Selection Using Firefly Algorithm in Hyperspectral Images for Classification. Int. J. Eng. Res. Technol. (IJERT).

[27] Ouf N.S. (2023). Leguminous seeds detection based on convolutional neural networks: Comparison of faster R-CNN and YOLOv4 on a small custom dataset. Artif. Intell. Agric..

[28] Chagas P., Akiyama R., Meiguins A., Santos C., Saraiva F., Meiguins B., Morais J. Evaluation of Convolutional Neural Network Architectures for Chart Image Classification. Proceedings of the International Joint Conference on Neural Networks.

[29] Książek K., Romaszewski M., Głomb P., Grabowski B., Cholewa M.J.S. (2020). Blood stain classification with hyperspectral imaging and deep neural networks. Sensors.

[30] Lehmann M.K., Gurlin D., Pahlevan N., Alikas K., Conroy T., Anstee J., Balasubramanian S.V., Barbosa C.C., Binding C., Bracher A.J.S.d. (2023). GLORIA-A globally representative hyperspectral in situ dataset for optical sensing of water quality. Sci. Data.

[31] Ghasemi M., kadkhoda Mohammadi S., Zare M., Mirjalili S., Gil M., Hemmati R. (2022). A new firefly algorithm with improved global exploration and convergence with application to engineering optimization. Decis. Anal. J..

